# QT Interval Prolongation Is a Novel Predictor of 1-Year Mortality in Patients With COVID-19 Infection

**DOI:** 10.3389/fcvm.2022.869089

**Published:** 2022-06-09

**Authors:** Ariel Banai, Yishay Szekely, Lior Lupu, Ariel Borohovitz, Erez Levi, Eihab Ghantous, Philippe Taieb, Aviram Hochstadt, Shmuel Banai, Yan Topilsky, Ehud Chorin

**Affiliations:** Department of Cardiology, Tel Aviv Sourasky Medical Center, Sackler School of Medicine, Tel Aviv University, Tel Aviv, Israel

**Keywords:** QTc interval, mortality, COVID-19, myocardial injury, ECG

## Abstract

**Background:**

QT interval prolongation is common in critically ill patients and is associated with increased mortality. However, the predictive value of a prolonged corrected QT interval (QTc) for myocardial injury and long-term mortality among patients hospitalized with COVID-19 infection is not well known.

**Purpose:**

To evaluate the association of prolonged QTc with myocardial injury and with 1-year mortality among patients hospitalized with COVID-19 infection.

**Materials and Methods:**

A total of 335 consecutive patients hospitalized with COVID-19 infection were prospectively studied. All patients underwent a comprehensive echocardiographic evaluation within 48 h from admission. Using the Bazett formula, the QTc interval was calculated from the first ECG tracing recorded at the ER. QTc ≥ 440 ms in males and ≥450 ms in females was considered prolonged. Patients with elevated cardiac biomarkers and/or echocardiographic signs of myocardial dysfunction were considered to have myocardial injury. The predictive value of QTc prolongation for myocardial injury was calculated using a multivariate binary regression model. One-year mortality rate of patients with and without QTc prolongation was compared using the log-rank test, and a multivariate Cox regression model adjusting for multiple covariates was performed to evaluate the 1-year mortality risk.

**Results:**

One-hundred and nine (32.5%) patients had a prolonged QTc. Compared to patients without QTc prolongation, patients with prolonged QTc were older (70 ± 14.4 vs. 62.7 ± 16.6, *p* < 0.001), had more comorbidities, and presented with a more severe disease. Prolonged QTc was an independent predictor for severe or critical disease (adjusted HR 2.14, 95% CI 1.3–3.5; *p* = 0.002) and myocardial injury (adjusted HR 2.07, 95% CI 1.22–3.5; *p* = 0.007). One-year mortality of patients with prolonged QTc was higher than those with no QTc prolongation (40.4% vs. 15.5; *p* < 0.001). Following adjustment to multiple covariates including myocardial injury and disease severity, QTc prolongation was found to be associated with increased 1-year mortality risk (HR 1.69, 95% CI 1.06–2.68, *p* = 0.027).

**Conclusion:**

Prolonged QTc is associated with disease severity, myocardial injury and 1-year mortality among patients hospitalized with COVID-19 infection.

## Introduction

The corrected QT interval (QTc) has been found to be a useful clinical tool for the identification of patients who are at a high risk of developing life-threatening ventricular arrhythmias and sudden cardiac death (1, 2). The assessment of the QTc is also useful for monitoring adverse effects of pharmacological agents that cause QTc prolongation and thus place individuals at high risk for sudden cardiac death (3, 4). Interestingly, QTc > 500 ms was also found to be associated with increased non-cardiovascular mortality (5), and the mechanism for this observation can only be speculated.

Evidence indicates that systemic inflammation, as reflected by CRP and IL-6 levels, is associated with an increased risk of malignant ventricular arrhythmias and SCD, both in patients with overt cardiac diseases and in apparently healthy subjects (6, 7). In accordance with these data, a significant relationship between QTc duration and systemic inflammatory activation, as assessed by CRP and cytokine levels, has been demonstrated in large populations of apparently healthy subjects, as well as in chronic inflammatory diseases, which frequently show QTc prolongation (8, 9).

Coronavirus disease 2019 (COVID-19) infection is associated with electrocardiographic abnormalities such as the prolongation in activation (QRS) and repolarization (QTc) (10, 11). COVID-19 infection is also associated with myocardial injury, indicated by both elevated cardiac biomarkers [Troponin and brain natriuretic peptide (BNP)] (12–15) and echocardiographic abnormalities (16). Previous studies described the association of QTc prolongation to myocardial injury and to short term mortality (17–19), and in the largest cohort consisting of 7,098 hospitalized COVID-19 patient, Fishbein et al. (20) reported that extremely prolonged QTc (>500 ms) was an independent predictor for in-hospital mortality. However, the association of prolonged QTc to long term mortality, had not yet been described. The objective of the current study is to test the hypothesis that a prolonged QTc in patients with COVID-19 is a marker for cardiac involvement in the disease, and may be an indicator to its severity, hereby providing valuable prognostic information toward the subsequent long term clinical course and mortality risk. This knowledge may guide initial triage decisions among infected patients and may help to identify high-risk patients.

## Materials and Methods

### Study Population

We prospectively studied 335 patients consecutively admitted in the Tel Aviv Sourasky Medical Center between 21 March 2020 and 16 September 2020. All Patients were diagnosed with COVID-19 infection confirmed by a positive reverse-transcriptase-polymerase chain reaction assay for SARS-CoV-2 obtained from respiratory tract sample. Demographic data, past medical history, medications, laboratory, and physical examination findings were recorded systematically. Patients’ clinical course, treatment, use of vasopressors and need for invasive/non-invasive ventilation were documented daily. All patients underwent a comprehensive echocardiographic and lung ultrasound evaluation within 48 h from admission, as part of a predefined step-by-step protocol, described in our previous publications (16, 21). Disease severity was assessed using National Institute of Health (NIH) risk stratification (asymptomatic: Individuals positive for SARS-CoV-2 but have no symptoms that are consistent with COVID-19; mild: signs and symptoms of COVID-19 excluding shortness of breath, dyspnea, or abnormal chest imaging; moderate: evidence of lower respiratory disease during clinical assessment or imaging and oxygen saturation ≥94% on room air; severe: oxygen saturation <94% on room air, arterial partial pressure of oxygen to fraction of inspired oxygen ratio <300 mmHg, respiratory frequency >30 breaths/min, or lung infiltrates >50%; critical: respiratory failure, septic shock, and/or multiple organ dysfunction) (22). Follow up data regarding mortality was obtained from the Israeli national ministry of health. The study was approved by the Tel Aviv Medical Center Ethics Committee (IRB number 0196-20-TLV).

### ECG Analysis

ECG tracing was performed upon arrival to the ER, prior to treatment administration. QT and QTc intervals were automatically performed by the ECG software [the Marquette 12 SL algorithm (GE Healthcare, Chalfont, United Kingdom) version 241 (150 Hz sampling rate) (MAC 5500)]. For quality assurance, ∼20% (67) of the QTc measurements obtained from the ECG software were validated by a senior cardiologist experienced in QT interval measurement, using the “tangent” method (23). This validation showed high agreement between automatically obtained and measured QTc intervals (Pearson correlation 0.94, *p* < 0.001). QTc was calculated from the QT and the R-R intervals using the Bazett formula and was considered prolonged if ≥440 ms in males and ≥450 ms in females. QTc ≥ 500 ms was considered extremely prolonged. For patients with atrial fibrillation the QTc was calculated manually using the following method: we calculated the QT intervals following the shortest and longest R-R intervals and divided each by the square root of the R-R interval preceding it. The average of these intervals was used as the QTc (24). The QRS interval was measured from the onset of the Q wave, or the R wave if no Q wave was visible, to the J point. The difference between the QTc and the QRS interval produced the JTc interval. For patients with wide a QRS complex (≥120 ms) due to either bundle branch block or cardiac pacing, QTc was calculated using the following formula: QTc = QTc-(QRS-120) (25).

### Echocardiographic Evaluation

The step-by-step protocol of the comprehensive echocardiographic evaluation was previously described (16).

### Outcomes

The primary outcome we evaluated was the association of QTc prolongation with 1-year mortality among hospitalized COVID-19 patients. Mortality rate and adjusted risk were calculated for the entire cohort, and also only among patients who survived the initial 30 days from admission. Additionally, we studied the association of QTc prolongation with a severe or critical disease severity and to myocardial injury at presentation. Patients whose initial laboratory tests showed either Troponin ≥50 ng/dL or BNP ≥82 pg/mL, as well as those whose echocardiographic evaluation revealed either left ventricular ejection fraction (LVEF) ≤ 50%, diastolic dysfunction grade ≥II, right ventricular dilatation or right ventricular reduced function, were considered to have myocardial injury.

### Statistical Analysis

Continuous variables were tested for normality using histograms, quantile-quantile plots, and the Shapiro–Wilk test. Normally distributed continuous variables were compared using the Student’s *t*-test and are presented as mean ± standard deviation, whereas non-normally distributed variables were compared using the Mann–Whitney *U* test and are presented as median and interquartile range. Categorial variables were compared using the Chi-Square test and the Fisher’s test and are presented as numbers and percentiles. A multivariate binary regression model was used to evaluate the association of QTc prolongation to disease severity and to myocardial injury, and a multivariate Cox regression model was used to evaluate the association of prolonged QTc to 1-year mortality. In the first step all variables considered for inclusion in the regression model were tested multicollinearity using correlation factor analyses. Variables that were highly correlated (correlation coefficient >0.7, *p* < 0.001) were considered to express multicollinearity and were excluded from the regression model. In all models, all baseline characteristics variables which differed between the two groups, namely, age, gender, ischemic heart disease, congestive heart failure hypertension, diabetes, hyperlipidemia, active malignancy, previous cerebrovascular accident or transient ischemic attack, atrial fibrillation or flutter, myocardial injury, and disease severity at admission were included in the model, and were then considered for removal using the Wald backward section method, with *p* > 0.1 set as a cut off for removal. Results of logistic regression models are reported as hazard ratios (HR) with their corresponding 95% confidence interval (CI). Comparison of 1-year mortality rate of patients with and without QTc prolongation was performed by the log-ranked test and is illustrated using Kaplan-Meier curve. For all calculations a *p*-value < 0.05 was considered significant. Statistical analysis was preformed using IBM SPSS Statistics for Windows, Version 28.0. Armonk, NY, United States, IBM Corp.

## Results

### Study Population

A total of 676 consecutive patients were admitted to the Tel Aviv Medical Center with COVID-19 infection between March 2020 and September 2020. One hundred and fifty-eight patients with no ECG tracing were excluded, as well as 183 patients who did not undergo the comprehensive echocardiography evaluation within 48 h from admission. The reasons for not completing the echocardiographic evaluation were: hospital discharge within 48 h from admission (37 patients), missing echocardiography data (48 patients) patient refusal (3 patients), death within 48 h from admission (80 patients) and patients with a “do not resuscitate/intubate status, designated for palliative care only (15 patients).” Hence, 335 patients were included in the final study cohort.

Patients baseline characteristics, laboratory test results and ECG findings are presented in Table 1. Two hundred and twenty-six patients had normal QTc, 109 patients had prolonged QTc, and 7 (2.1%) had QTc ≥ 500 ms. Mean age was 65.1 ± 16.13 and 202 (60.3%) were males. Patients with QTc prolongation were older (70 ± 14.4 vs. 62.7 ± 16.6, *p* < 0.001), and were more likely to have ischemic heart disease, hypertension, hyperlipidemia, diabetes mellitus, chronic renal failure, and a history of cerebrovascular accident/transient ischemic attack. Additionally, baseline medical treatment with aspirin, ACEI/ARB, beta-blocker and metformin was more frequent amount patients with prolonged QTc. Compared to patients without QTc prolongation, patients with prolonged QTc had a higher disease severity on presentation, as well as higher levels of Troponin-I, BNP, and C-reactive protein.

**TABLE 1:** Baseline characteristics.

	All *n* = 335	Normal QTc *n* = 226	prolonged QTc *n* = 109	*p*
Age, years	65.1 ± 16.13	62.7 ± 16.6	70 ± 14.4	<0.001
Gender, male (%)	202 (60.3)	128 (56.6)	74 (67.9)	0.057
Body mass index, Kg/m^2^	27.6 ± 5.9	27.2 ± 6.1	28.6 ± 5.9	0.074
Obesity, *n* (%)	86 (25.7)	52 (23)	34 (31.2)	0.112
Ischemic heart disease, *n* (%)	58 (17.3)	28 (12.4)	30 (27.5)	0.001
Congestive heart failure, *n* (%)	32 (9.6)	17 (7.5)	15 (13.8)	0.076
Atrial fibrillation, *n* (%)	36 (10.7)	19 (8.4)	17 (15.6)	0.059
Cerebrovascular accident/transient ischemic attack, *n* (%)	31 (9.3)	15 (6.6)	16 (14.7)	0.026
Peripheral vascular disease, *n* (%)	12 (3.6)	5 (2.2)	7 (6.4)	0.064
Chronic renal failure, *n* (%)	38 (11.3)	17 (7.5)	21 (19.3)	0.003
Diabetes mellitus, *n* (%)	119 (35.5)	64 (28.3)	55 (50.5)	<0.001
Hypertension, *n* (%)	177 (52.8)	104 (46)	73 (67)	<0.001
Hyperlipidemia, *n* (%)	131 (39.1)	78 (34.5)	53 (48.6)	0.017
Malignancy, *n* (%)	18 (5.4)	9 (4)	9 (8.3)	0.123
**Disease severity**				
Asymptomatic disease, *n* (%)	15 (4.5)	12 (5.3)	3 (2.8)	0.402
Mild disease, *n* (%)	70 (20.9)	54 (23.9)	16 (14.7)	0.062
Moderate disease, *n* (%)	71 (21.2)	55 (24.3)	16 (14.7)	0.046
Severe disease, *n* (%)	124 (37)	75 (33.2)	49 (45)	0.041
Critical disease, *n* (%)	55 (16.4)	30 (13.3)	25 (22.9)	0.028
Hydroxychloroquine/Azenil, *n* (%)	53 (15.8)	36 (15.9)	17 (15.6)	1
Laboratory myocardial injury, *n* (%)	96 (28.7)	50 (22.1)	46 (42.2)	<0.001
Echocardiographic myocardial injury, *n* (%)	159 (47.5)	92 (40.7)	67 (61.5)	<0.001
Myocardial injury, *n* (%)	188 (56.1)	110 (48.7)	78 (71.6)	<0.001
**Laboratory test results**				
White blood cells, 10^3^/μL, median [IQR]	6.7 [5.1–8.9]	6.5 [5–8.6]	6.9 [5.6–9.8]	0.021
Hemoglobin, median [IQR], g/dL	13.2 ± 2	13.7 [12.5–14.7]	13.4 [11.5–14.6]	0.19
Platelets, median [IQR], 10^3^/μL	186 [147–248]	186 [146–249]	193 [150–247]	0.45
Creatinine, median [IQR], mg/dL	0.89 [0.71–1.16]	0.87 [0.7–1.1]	0.98 [0.76–1.4]	0.008
Blood urea nitrogen, median [IQR], mg/dL	17 [12–24]	16 [12–22]	20 [14–29]	<0.001
Sodium, mean ± SD, mmol/L	135.8 ± 3.9	135.9 ± 3.6	135.7 ± 4.5	0.283
Potassium, mean ± SD, mmol/L	4.03 ± 0.57	4.04 ± 0.58	4.01 ± 0.56	0.644
Magnesium, mean ± SD, mg/dL	2.03 ± 0.3	2.04 ± 0.3	2 ± 0.2	0.437
Calcium, mean ± SD, mg/dL	8.62 ± 0.58	8.71 ± 0.5	8.43 ± 0.62	<0.001
Troponin-I, ng/dL, median [IQR]	11 [5–24]	6 [3–16]	18 [8–43]	<0.001
Troponin >50 ng/L, *n* (%)	33 (9.9)	12 (5.3)	21 (19.4)	<0.001
Brain natriuretic peptide, pg/mL, median [IQR]	62 [25–196]	44.5 [16–108]	97 [33–286]	<0.001
BNP >82 pg/mL *n* (%)	86 (37.7)	47 (30.3)	39 (53.4)	0.001
D-dimer, mg/L, median [IQR]	1 [0.6–1.7]	0.83 [0.5–1.7]	1.1 [0.64–2]	0.066
D-dimer >0.5 mg/L, *n* (%)	237 (75.9)	154 (0.7)	83 (80.6)	0.206
C-reactive protein, mg/L, median [IQR]	75 [30–147]	60 [21–136]	86 [29–158.2]	0.02
CRP >5 mg/L, *n* (%)	310 (93.1)	207 (92)	103 (95.4)	0.356
Fibrinogen, mg/dL, mean ± SD	535 ± 152.4	535.3 ± 159.3	534.1 ± 138.9	0.95
Fibrinogen >470 mg/dL, *n* (%)	181 (62.6)	118 (61.8)	63 (64.3)	0.702
**Treatment**				
Aspirin	84 (25.1)	48 (21.2)	36 (33)	0.023
P2Y_12_ inhibitor	25 (7.5)	14 (6.3)	11 (10.1)	0.266
Non-vitamin K anticoagulation	34 (10.1)	18 (8)	16 (14.7)	0.081
Angiotensin-converting enzyme/Angiotensin-receptor blocker	131 (39.1)	76 (33.6)	55 (50.5)	0.004
Angiotensin receptor-neprilysin inhibitor	1 (0.3)	0 (0)	1 (0.9)	0.325
Beta-blocker	107 (31.9)	55 (24.3)	52 (47.7)	<0.001
Metformin	76 (22.7)	43 (19)	33 (30.3)	0.026
Sodium-glucose cotransporter inhibitor	11 (3.3)	5 (2.2)	6 (5.5)	0.186
Steroids	22 (6.6)	16 (7.1)	6 (5.5)	0.647

Patients baseline ECG characteristics are presented in Table 2. Compared to patients without QTc prolongation, patients with prolonged QTc had higher frequency of atrial fibrillation, higher heart rate, and were more often tachycardic. Among patients with prolonged QTc bundle branch block was more common among, and QRS duration was longer.

**TABLE 2:** ECG characteristics.

	Normal QTc *n* = 226	Prolonged QTc *n* = 109	*p*
Sinus rhythm, *n* (%)	214 (94.7)	96 (88.1)	0.044
Atrial fibrillation, *n* (%)	10 (4.4)	13 (11.9)	0.019
Heart rate, beats/minute, mean ± SD	81.2 ± 16.4	87.1 ± 19.5	0.007
Heart rate >100 beats/min	29 (12.8)	28 (25.7)	0.005
QRS, ms, median [IQR]	86 [78–96]	95 [84–117.5]	<0.001
QT, ms, mean ± SD	368.8 ± 38.2	399.6 ± 54.8	<0.001
QTc ms, median [IQR]	418.5 [403–432]	459 [451–479.5]	<0.001
JTc, ms, mean ± SD	327.4 ± 24	368.9 ± 23.5	<0.001
RBBB, *n* (%)	11 (4.9)	14 (12.8)	0.014
LBBB, *n* (%)	1 (0.4)	6 (5.5)	0.006

*IQR, interquartile range; RBBB, right bundle branch block; LBBB, left bundle branch block.*

### Disease Severity

In a multivariate Binary regression model prolonged QTc was the strongest predictor for a severe or critical disease at presentation (HR 2.1 95% CI 1.31–3.5; *p* = 0.002), followed by age (HR 1.02, 95% CI 1.01–1.03; *p* = 0.005) (Supplementary Table 1).

### Myocardial Injury

**FIGURE 1:**
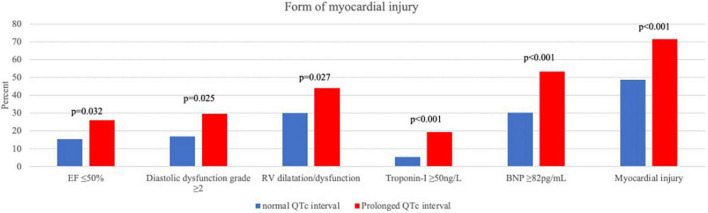
Forms of myocardial injury.

Myocardial injury, whether in the form of elevated cardiac biomarkers (Troponin-I and BNP) or in the form of an abnormal echocardiography examination, was more frequent among patients with prolonged QTc (Figure 1). In a multivariate binary regression model (Supplementary Table 2), QTc prolongation was associated with a twofold increased risk for myocardial injury (HR 2.07, 95% CI 1.22–3.5; *p* = 0.007). Congestive heart failure and age were also associated with increased risk for myocardial injury (HR 5.28 95% CI 1.5–18.6; *p* = 0.01 and HR 1.04 95% CI 1.03–1.06; *p* < 0.001, respectively).

### Outcome Analysis

**FIGURE 2:**
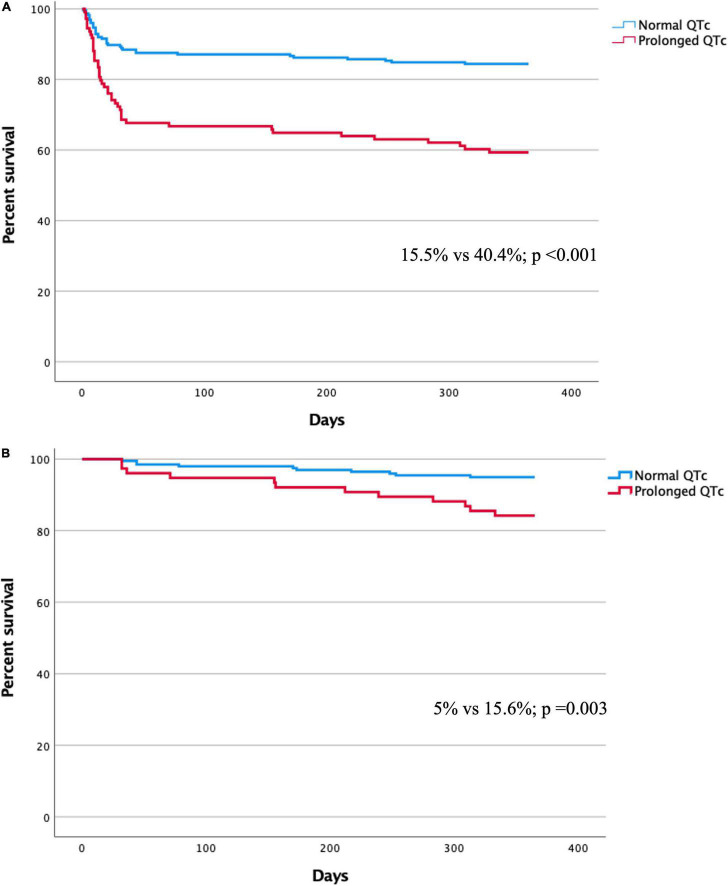
**(A)** Kaplan-Meier curve for 1-year mortality in patients with and without QTc prolongation. **(B)** Kaplan-Meier curve for 1-year mortality among 30-day survivors in patients with and without QTc prolongation.

During the 1-year follow-up, the mortality rate of patients with QTc prolongation was higher compared to those with no QTc prolongation [44/109 (40.4%) vs. 35/226 (15.5%); *p* < 0.001] (Figure 2A). In a multivariate Cox regression analysis, the adjusted 1-year mortality risk was 69% higher in patients with QTc prolongation (adjusted HR 1.69, 95% CI 1.06–2.68; *p* = 0.027) (Table 3). Analysis preformed among 30-day survivors revealed that the 1-year mortality rate as well as the adjusted 1-year mortality risk remained significantly higher in patients with prolonged QTc at presentation compared to patients without QTc prolongation [12/77 (15.6%) vs. 10/200 (5%); *p* = 0.03, adjusted HR 2.42, 95% CI 1.02–5.74; *p* = 0.044, respectively] (Figure 2B and Table 3). Compared to patient without QTc prolongation and no myocardial injury, the adjusted 1-year mortality risk was highest in patients with prolonged QTc and myocardial injury (HR 6.63, 95% CI 2.28–19.3; *p* = 0.001), followed by patients with QTc prolongation without myocardial injury (HR 6.12 95% CI 1.83–20.49; *p* = 0.003), and patients with myocardial injury without QTc prolongation (HR 4.95 95% CI 1.83–20.49; *p* = 0.003) (Table 4).

**TABLE 3:** Multivariate Cox regression for 1-year mortality.

	All patients				30-day survivors			
	95% confidence interval				95% confidence interval			
	HR	Lower	Higher	*p*	HR	Lower	Higher	*p*
Age	1.066	1.045	1.088	0.000	1.107	1.063	1.152	<0.001
Congestive heart failure	2.298	1.353	3.905	0.002				
Hyperlipidemia	0.599	0.380	0.944	0.027	0.297	0.117	0.751	0.01
Disease severity	1.441	1.150	1.807	0.002				
Myocardial injury	2.238	1.168	4.287	0.015	2.861	0.82	9.985	0.099
Prolonged QTc	1.687	1.062	2.679	0.027	2.424	1.022	5.746	0.044

*HR, hazard ratio.*

**TABLE 4:** Multivariate Cox regression for 1-year mortality stratified by QTc prolongation and myocardial injury.

	HR	95% confidence interval		*p*
		Lower	Higher	
Age	1.065	1.044	1.087	< 0.001
Congestive heart failure	2.337	1.377	3.966	0.002
Hyperlipidemia	0.582	0.370	0.917	0.02
Disease severity	1.476	1.174	1.856	0.001
Prolonged QTc (−), myocardial injury (−)	Reference			
Prolonged QTc (+), myocardial injury (−)	6.122	1.829	20.495	0.003
Prolonged QTc (−), myocardial injury (+)	4.954	1.715	14.316	0.003
Prolonged QTc (+), myocardial injury (+)	6.631	2.278	19.305	0.001

*HR, hazard ratio.*

Seven patients (2.1%) were found to have extremely prolonged QTc ≥ 500 ms. Six out of these seven patients (85%) had myocardial injury, and the 1-year mortality was 71% (5 patients).

## Discussion

Time from the QRS onset to the T-wave end, the QT interval, is a measure of repolarization duration and is clinically used to detect the short and long QT syndromes as well as to assess disease and drug related repolarization effects. According to several epidemiologic studies in the general population, QT-interval prolongation is, at the most, a weak risk factor of mortality (26, 27). In the Strong Heart Study, Bazett-corrected QT interval was a stronger independent predictor of all-cause mortality (28). Stress-induced cardiomyopathy is reported in as many as 25% of patients hospitalized in medical intensive care units because of serious infections (29), and this form of cardiomyopathy is invariably associated with QT prolongation (30).

To the best of our knowledge, this is the first study evaluating the association of prolonged QTc to myocardial injury and 1-year mortality in consecutively hospitalized patients with COVID-19 infection. Importantly, while previous studies evaluating the association of prolonged QTc to myocardial injury and mortality, defined myocardial injury by means of elevated cardiac biomarkers, namely Troponin and BNP, in our study, the myocardial injury was assessed by both cardiac biomarkers as well as a comprehensive echocardiographic evaluation performed in all patients within 48 h from admission.

Our main finding is that among patients hospitalized with COVID-19 infection, QTc prolongation at admission is predictive of disease severity, myocardial injury and 1-year mortality. There is not a single QTc value that tells apart all healthy individuals from all patients affected by a congenital long QT syndrome. Instead, the QTc of the healthy population and that of patients with long QT syndrome, both have a normal distribution with significant overlap between the two curves. The QTc of women is longer than that of men but that is true for healthy individuals and for patients affected by a long QT syndrome. A QTc of <400 ms for males (QTc < 420 ms for females) is considered “normal” because such values are close to the 50th percentile of the QTc of healthy individuals. We found that even mild QT prolongation (QTc > 440 ms for males and QTc > 450 ms for females) *at baseline* is predictive of disease severity, myocardial injury and 1-year mortality. Importantly, we found that QTc prolongation was associated with 1-year mortality also among patients surviving the first 30 days of hospitalization, underscoring the predictive value of QTc for long-term mortality.

The QTc values above relate only to patients with narrow QRS complexes. For patients with wide a QRS complex due to either bundle branch block or cardiac pacing, a JTc (defined as QTc – QRS) should be considered “prolonged” if >360 ms for males and or 370 ms for females (31). Extreme QT values (QTc > 500 ms) roughly correspond to JTc > 410 ms for patients with wide QRS complex. We show that the effect on the QTc interval was driven entirely by prolonging repolarization and regardless of QRS duration, as evident by the corresponding JTc interval prolongation.

Several recent studies have shown the association of prolonged QTc to increased mortality in hospitalized COVID-19 patients (32–35). Thakore et al. (32) have reported that prolonged QTc on admission was associated with increased mortality, a finding that remained significant following adjustment to multiple covariates, including cardiac involvement, defined as elevated cardiac biomarkers. Our study is the first to report the association of QTc prolongation to myocardial injury, defined as either cardiac biomarkers elevation or echocardiographic abnormalities, in a cohort of consecutively hospitalized COVID-19 patients. Defining myocardial injury using both cardiac biomarkers elevation and imaging may have resulted in the inclusion of patients which may have been inappropriately classified as not having myocardial injury in previous studies. Indeed, in our cohort, 80 patients (58.8%) with echocardiographic abnormalities, had no elevation in cardiac biomarkers. This finding underscores the importance of echocardiography evaluation as an integral part of risk stratification among hospitalized COVID-19 patients.

Interestingly, our finding of higher mortality risk in patients with prolonged QTc was also evident among the subpopulation of patients with no myocardial injury. While our study can not explain this observation, a potential explanation could be that QTc prolongation reflects a subtle and early sign of myocardial dysfunction, not yet evident as cardiac biomarkers elevation or echocardiographic abnormalities.

Whereas the association of active inflammation to QTc prolongation and arrhythmias has been previously described (8, 9, 36, 37), the COVID-19 pandemic provided an exceptional opportunity to further investigate this association in the setting of an acute, systemic, infective disease. Severe COVID-19 disease is characterized by an unopposed cytokine activation leading to an unregulated inflammatory response which is associated with higher morbidity and mortality (38), and the role played by proinflammatory cytokines in the prolongation of the QTc is becoming increasingly recognized (39–41). Lazzerini et al. (42) have recently reported that among patients with severe COVID-19 infection and elevated IL-6, regardless of acute myocardial injury and concomitant QT-prolonging risk factors, QTc was significantly prolonged. These observations suggest monitoring QTc and actively counteracting measures that may prolong it, may be beneficial in patients with COVID-19 infection.

Our study has several limitations. Firstly, it is a single center observational study, and an inherent selection bias can not be excluded. “Secondly, in our cohort, patients with prolonged QTc were older, had a higher comorbidity burden and presented more frequently with a severe or critical COVID-19 infection. Although adjustment for these differences was performed, our results may be influenced by other unaccounted confounders. Therefore, our observations should be interpreted cautiously.” Thirdly, mortality data was obtained from a national digital registry, and the cause of death could not be accounted for. Fourthly, since most of the patients did not have a baseline echocardiography, it could be argued that some were inappropriately classified as having acute COVID-19 related myocardial injury. In our cohort, 9.6% of patients had a history of heart failure. Additionally, 10.7% had a history of atrial fibrillation and 17.3% had a history of ischemic heart disease, both of which might be associated with subtle echocardiographic abnormalities. Therefore, it is possible that some of the echocardiographic abnormalities reported reflect preexisting comorbidities rather than an acute myocardial injury. Fifthly, in most patients, QTc interval was automatically obtained from the ECG software. Although we found very high correlation between the automatically obtained and manually measured QTc intervals during the validation process, computer errors can not be excluded. Additionally, the QTc interval was calculated using the Bazett formula, which is known for overestimation in patients with tachycardia (43–45). In our study 57 patients (17%) had a heart rate ≥100 in their baseline ECG, and therefore, their QTc may be overestimated. Lastly, as our cohort included only hospitalized patients, thus the applicability of our findings to COVID-19 outpatients can not be determined from this study.

## Conclusion

Prolonged QTc, even when mild, is an independent risk factor for disease severity, myocardial injury and 1-year mortality among patients hospitalized with COVID-19 infection. Our finding suggests that the QTc interval could assist in the identification of high-risk patients, and its role in the risk stratification of hospitalized COVID-19 patients should be further investigated.

## Data Availability Statement

The raw data supporting the conclusions of this article will be made available by the corresponding author upon reasonable request.

## Ethics Statement

The studies involving human participants were reviewed and approved by the IRB number 0196-20-TLV. Written informed consent for participation was not required for this study in accordance with the National Legislation and the Institutional Requirements.

## Author Contributions

ABa: writing—original draft and formal analysis. YS: conceptualization and methodology. LL, ABo, EL, EG, and PT: project administration. AH: writing—reviewing and editing and formal analysis. SB and YT: supervision. EC: supervision, methodology, and writing—reviewing and editing. All authors contributed to the article and approved the submitted version.

## Conflict of Interest

The authors declare that the research was conducted in the absence of any commercial or financial relationships that could be construed as a potential conflict of interest.

## Publisher’s Note

All claims expressed in this article are solely those of the authors and do not necessarily represent those of their affiliated organizations, or those of the publisher, the editors and the reviewers. Any product that may be evaluated in this article, or claim that may be made by its manufacturer, is not guaranteed or endorsed by the publisher.
