# Supplementary material for: QT Interval Prolongation Is a Novel Predictor of 1-Year Mortality in Patients With COVID-19 Infection
Source: Front Cardiovasc Med. 2022 Jun 9;9:869089. doi: 10.3389/fcvm.2022.869089 (PMC9223350; doi:10.3389/fcvm.2022.869089)
Supplement: Supplementary file 1 [file Data_Sheet_1.pdf]

Supplementary Table 1: Binary regression for Disease severity

	HR	95% confidence interval		p
		Lower	Higher	
Age	1.02	1.01	1.03	0.005
Prolonged QTc	2.14	1.31	3.49	0.002

Abbreviations: HR: hazard ratio

Supplementary Table 2: Binary regression for myocardial injury

	HR	95% confidence interval		p
		Lower	Higher	
Age	1.042	1.026	1.059	<0.001
Prolonged QTc	2.070	1.223	3.505	0.007
Congestive heart failure	5.285	1.498	18.644	0.010

Abbreviations: HR: hazard ratio
